# Machine Learning-Based Model for Grip Strength Prediction in Healthy Adults: A Nationwide Dataset-Based Study

**DOI:** 10.3390/jcm14051542

**Published:** 2025-02-25

**Authors:** Mina Park, Yeo Hyung Kim, Jung Soo Lee

**Affiliations:** Department of Rehabilitation Medicine, College of Medicine, The Catholic University of Korea, Seoul 06591, Republic of Korea; mmaabb0822@gmail.com (M.P.); drkyh@catholic.ac.kr (Y.H.K.)

**Keywords:** handgrip strength, machine learning, XGBoost model, multilayer perceptron

## Abstract

**Background**: This study aimed to develop machine learning models for estimating the handgrip strength (HGS) in healthy adults and to identify the model with the highest accuracy and generalizability. **Methods**: Data from the Korean National Health and Nutrition Examination Survey (2014–2019), including 21,147 participants aged >19 years, were analyzed. The maximum HGS was measured using a standardized protocol, with 11 demographic, anthropometric, and physical activity predictors. Polynomial regression (PR), multilayer perceptron (MLP), and extreme gradient boosting (XGBoost) models were developed and evaluated using the root-mean-square error (RMSE) and coefficient of determination (R^2^). **Results**: The HGS was found to vary by gender, age, and hand dominance, with males and younger individuals showing higher values. The XGBoost model achieved the highest R^2^ (0.717), demonstrating superior predictive accuracy and generalizability compared with PR and MLP. Key predictors in the XGBoost model included weight, age, height, and waist circumference, while hand dominance was less significant. **Conclusions**: The XGBoost model outperformed the MLP and PR models, achieving the highest R^2^ value. It holds promise for clinical applications, enabling accurate HGS estimation to support early diagnosis, targeted interventions, and personalized goal setting.

## 1. Introduction

Hand grip strength (HGS) serves as a fundamental indicator of overall muscular strength and function. This simple yet robust measure has garnered increasing attention in clinical research because of its multifaceted implications for health and well-being [1]. Its clinical significance extends beyond its role in assessing physical performance, encompassing a range of health-related domains [2]. Numerous studies have demonstrated the association between HGS and various health outcomes. Reduced HGS has been linked to increased mortality associated with medical diseases, cardiovascular events, and functional decline in the elderly [3,4]. Additionally, a growing body of evidence suggests a correlation between metabolic health, musculoskeletal disorders, and mental well-being [5,6,7]. HGS is also used as a diagnostic criterion for sarcopenia: a condition associated with physical disability, falls, and dependence on others in old age [8].

The simplicity and cost-effectiveness of the HGS assessment makes it an attractive diagnostic tool in various clinical settings to enhance the early detection of health issues and to facilitate timely interventions. Additionally, HGS can be used to assess clinical outcomes and to monitor the effectiveness of interventions [2,9]. As HGS can be used for a wide variety of clinical diagnoses and assessments, establishing accurate reference values is critical.

Although HGS can be easily measured, it exhibits high inter- and intra-individual variability. Furthermore, it is influenced by anthropometric parameters such as age, gender, weight, and height, and it is also affected by factors such as occupation and physical activity [10]. Although previous studies have attempted to estimate the reference values of HGS [11,12], they have been limited by insufficient sample sizes. Additionally, conventional statistical models, which assume a linear relationship between HGS and covariates, have limitations in representing a generalized regression model for HGS and may reduce predictive power owing to the potential for nonlinear relationships between HGS and these variables.

Therefore, the primary aim of this study was to build appropriate machine learning models to estimate the HGS in healthy adults and to identify the model with the highest predictive accuracy and generalizability.

## 2. Materials and Methods

### 2.1. Participants

This study adopted datasets from the sixth, seventh, and eighth Korean National Health and Nutrition Examination Survey (KNHNES VI-VIII), which was conducted from 2014 to 2019. These surveys, conducted annually by the Division of Chronic Disease Surveillance, Korean Centers for Disease Control and Prevention (KCDC), provide representative data on the health, nutritional status, and physical activity levels of the general Korean population. This study included 21,147 healthy participants aged over 19 years. “Healthy” was defined as having no limitations on daily and social activities, and it is indicated by a score of 1 on the EuroQol 5-Dimension Questionnaire (EQ-5D) index. The EQ-5D is a standardized, self-reported questionnaire developed by the EuroQol Group to assess health status.

### 2.2. Experimental Protocol

After KCDC obtained informed consent from all of the participants, they were interviewed and assessed by well-trained examiners using standardized physical examinations and protocols. Detailed information regarding the survey is available on the KNHANES website (https://knhanes.kdca.go.kr/knhanes/), accessed on 1 February 2024.

Maximum HGS (kg) was measured thrice alternately for both hands using a digital grip strength dynamometer (Takei Digital Grip Strength Dynamometer Model TKK5401; TAKEI, Niigata, Japan) following a standardized protocol. Eleven potential independent variables were included based on a previous study estimating grip strength: age, gender, hand preference, height, weight, waist circumference, and five physical activity levels (vigorous/moderate work, vigorous/moderate recreation, and travel). The physical activity levels were calculated using a validated Korean version of the World Health Organization Global Physical Activity Questionnaire (GPAQ).

The time spent engaging in moderate-to-vigorous physical activity was obtained from the datasets based on the frequency (days/week) and duration (min/day) of physical activity in a typical week. Activity intensity was expressed in metabolic equivalents of tasks (METs) (MET-min/week), which were calculated by multiplying the time spent in physical activity (min/week) by 4.0 and 8.0 METs for moderate and vigorous activities, respectively. The total moderate-to-vigorous physical activity was categorized into three levels (high, moderate, or low) according to the GPAQ analysis guide.

“Unknown” or “Non” responses were considered missing values, and participants with any missing values were excluded from this study.

### 2.3. Data Analysis 

In data preprocessing, the average of the three maximum HGS measurements was used for each participant to account for inter- and intra-individual variability. A robust scaler and log transformation were employed to minimize the influence of outliers and variance.

A grip strength estimation model was built using three different models: polynomial regression (PR), multilayer perceptron (MLP), and extreme gradient boosting (XGBoost). These models were selected to compare traditional statistical inference methods with modern machine learning-based predictive models, each possessing distinct characteristics with specific advantages and limitations.

PR is a form of regression analysis that models the relationship between independent and dependent variables as an nth-order polynomial. This approach allows for greater flexibility by increasing the polynomial degree when a linear relationship between the variables does not fully fit the data [13,14]. It is widely used as a reference model due to its ease of interpretation and simple implementation. However, it has limitations in capturing complex nonlinear relationships and is prone to overfitting with higher-degree polynomials.

Deep-learning models, such as MLP, use artificial neural networks to mimic the learning processes of the human brain. They comprise an input layer, hidden layers, and an output layer. MLP is particularly effective in capturing nonlinear patterns and automatically extracting features, making it highly suitable for complex datasets. However, it has a black-box nature, requires large datasets for optimal performance, and can be computationally expensive. In this study, the deep-learning model comprised hidden layers with 256 nodes using the tanh activation for nonlinearity. Additionally, batch normalization was employed for faster and more stable learning, and dropout layers with a ratio of 0.2 were used to block the signal from going to the node of the next layer and to prevent overfitting. An Adam optimizer with a learning rate of 0.001 was used for training. The batch size was set to 128, and the model was trained using the root-mean-square error (RMSE) as the loss function. To prevent overfitting and underfitting, training was limited to 100 epochs, with early stopping if the validation loss did not decrease for more than 10 epochs.

XGBoost is a gradient boosting framework designed for tree-based models [15,16]. It primarily uses decision trees as base learners. Decision trees are nonparametric supervised learning methods that are used for nominal or continuous outcomes. The gradient boosting framework of XGBoost comprises an ensemble learning technique that sequentially adds weak learners to minimize the loss function through decision trees. XGBoost is optimized for predictive performance, offering advantages such as built-in missing value handling, overfitting prevention, and strong generalization. However, it requires careful hyperparameter tuning and can be challenging to interpret. In this study, hyperparameters were tuned for optimal performance with the following settings: alpha = 2.5; booster = dart; subsample ratio of columns = 0.8; gamma = 0.5; lambda = 3; learning rate = 0.1; maximum tree depth = 3; and number of trees = 100.

The selection of these models was based on their methodological differences and practical relevance. We aimed to compare a traditional inference-based method (PR) with recent machine learning-based prediction models to evaluate their effectiveness in HGS prediction. Among various machine learning approaches, we selected MLP for its established effectiveness in previous research [17] and XGBoost for its superior predictive performance in recent machine learning applications [15]. 

The RMSE and coefficient of determination (R^2^) were used as evaluation metrics to assess the performance of the machine-learning models. The machine learning model in this study was implemented using Python 3.8.9. The following machine learning libraries were employed: TensorFlow 2.13.1, Keras 2.13.1, scikit-learn 1.3.2, and XGBoost 2.0.3.

## 3. Results

### 3.1. Baseline Characteristics of the 21,147 Participants

The analysis included 21,147 participants. The average maximum HGS (kg) and standard deviation were 29.7 ± 10.2 kg for the first measurement, 30.7 ± 10.2 kg for the second, and 30.9 ± 10.2 kg for the third. Across the three measurements, the inter-individual range of average HGS was 5.3–70.6 kg, with a standard deviation range of 0–20.9 kg.

**Figure 1:**
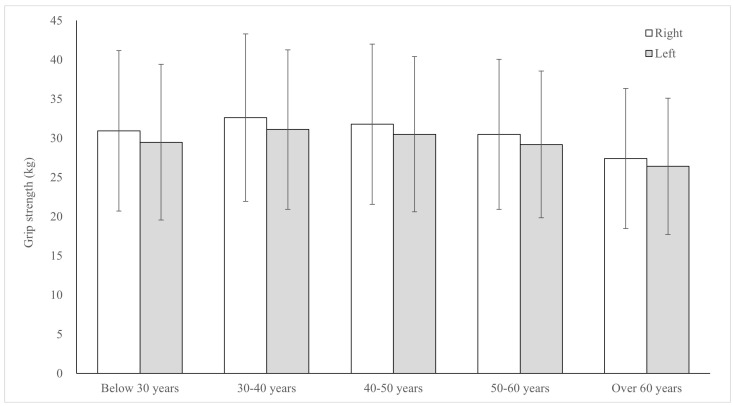
The average and standard deviation of grip strength according to age level.

The mean right/left hand maximum grip strength was 38.48 ± 7.69 kg/37.04 ± 7.28 kg for male participants and 22.97 ± 4.90 kg/21.83 ± 4.60 kg for female participants. In both males and females, the right-hand maximum grip strength was higher than that in the left hand. The mean right/left hand maximum grip strength, stratified by age group, was as follows: 30.92 ± 10.23 kg/29.47 ± 9.93 kg for those aged below 30 years; 32.62 ± 10.68 kg/31.11 ± 10.17 kg for those aged 30–40 years; 31.78 ± 10.23 kg/30.49 ± 9.90 kg for those aged 40–50 years, 30.49 ± 9.56 kg/29.19 ± 9.36 kg for those aged 50–60 years; and 27.42 ± 8.93 kg/26.42 ± 8.69 kg for those aged over 60 years (Figure 1). The raw data demonstrated inter- and/or intra-individual variations according to gender and age (Table 1).

### 3.2. Performance Comparisons of the Three Machine Learning Models

The best RMSE was 5.355 for a third-order PR model, including only anthropometric variables (excluding the physical activity levels), and 5.360 for the XGBoost model, using both anthropometric variables and physical activity levels (Table 2).

The best R^2^ values were 0.714 and 0.717 for the XGBoost model using anthropometric variables with and without the five physical activity levels. In terms of generalizability, the XGBoost model demonstrated the best ability to accurately predict grip strength.

**Figure 2:**
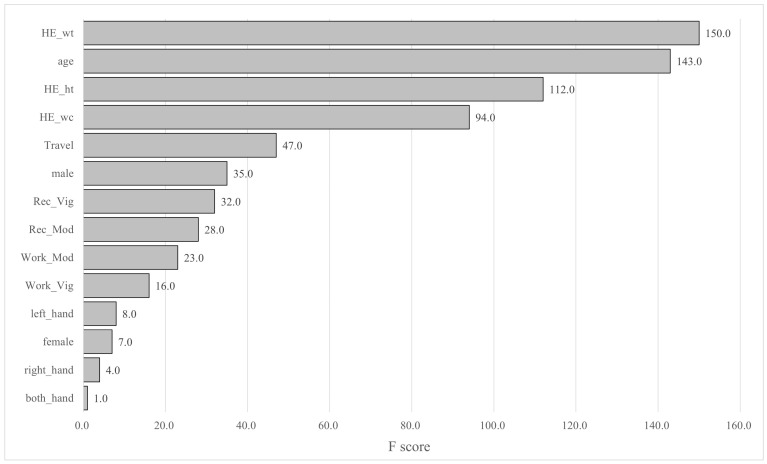
The importance of independent variables in the XGBoost model (F-score). Abbreviations: HE_wt: weight, HE_ht: height, HE_wc: waist circumference, Rec_vig: vigorous recreation activity, Rec_Mod: moderate recreation activity, Work_Mod: moderate work activity, and Work_Vig: vigorous work activity.

XGBoost offers several methods for evaluating variable importance in tree splitting, one of which is F-score. This metric is calculated by counting the number of times each feature is used to split the data across all trees, thereby providing a relative measure of importance. In this study, weight, age, height, and waist circumference were identified as the most important variables by F-score, while the dominant hand was the least critical (Figure 2).

## 4. Discussion

HGS is increasingly recognized as a vital sign indicative of an individual’s overall health status. Diminished HGS has been associated with higher risks of cardiovascular events, including heart attacks, and it is predictive of all-cause mortality [18]. Moreover, HGS is a key diagnostic criterion for sarcopenia, a critical health concern in the elderly [8]. This is because HGS serves as an indicator of overall muscle strength and physical function. Low HGS is associated with functional impairment, chronic disease, and increased mortality in older adults [3,4]. The European Working Group on Sarcopenia in Older People 2 (EWGSOP2) guidelines highlight the clinical relevance of HGS by setting diagnostic thresholds for sarcopenia at <27 kg for men and <16 kg for women [8].

In clinical practice, a routine assessment of HGS can facilitate the early identification of individuals at risk for adverse health outcomes. However, a direct measurement of an individual’s HGS requires specialized equipment, time, and a skilled examiner. In addition, results can vary depending on the measurement environment and posture. Estimating HGS using machine learning models trained on large datasets can mitigate these limitations in clinical settings, providing a quick and convenient assessment of HGS.

However, HGS estimation, when using classical statistical models, has its challenges owing to limitations such as a lack of representative data and the influence of potential confounding variables [12,19,20]. Moreover, traditional linear regression models, or its extended version (which was used in a previous study [21]), and even PR [19] are still classified as linear models. These models can only explain linear sections in two dimensions and are limited in their ability to capture the complex, nonlinear relationships between HGS and its predictors. This limitation can be overcome by machine learning models, which can analyze high-dimensional data and model linear relationships.

Machine learning approaches have been successfully applied in various medical fields, achieving exceptional and sometimes groundbreaking performance in prediction and classification tasks [21,22,23]. In this study, we developed machine learning models using a dataset of 21,147 healthy adults and explored the performance of three different models—multilayer perceptron (MLP), XGBoost, and PR—to determine the best HGS prediction model.

The results demonstrate that XGBoost outperformed the other models in predicting HGS, achieving an RMSE of 5.360 and R^2^ of 0.717, and it offered better generalizability. XGBoost has become increasingly popular in recent years, particularly in machine learning competitions and industrial applications. Its superior performance can be attributed to several factors. First, HGS is affected by many potential cofounding variables and exhibits nonlinear relationships with them [12,19,24]. XGBoost can handle both numerical and categorical data, as well as missing values, and it can be tuned to optimize performance for specific datasets using a rich set of hyperparameters. It works by combining multiple decision trees, and each decision tree makes a prediction based on a set of rules that can be easily visualized and understood. This makes XGBoost more interpretable, allowing data scientists to gain insights into the underlying relationships between outcomes and independent variables. Moreover, it provides feature importance scores that indicate the relative contribution of each feature to its predictions. This information is invaluable for feature selection and understanding the driving factors behind the outcomes.

Interestingly, the fourth-order PR model, when using both anthropometric variables and five physical activity levels, achieved the lowest RMSE of 4.989 compared to the other PR models without the five physical activity levels. However, it exhibited poor generalizability, with a test dataset RMSE of 16.624. This finding highlights that increasing model complexity by including more variables and higher-order terms does not necessarily improve the generalizability of the model.

Compared to a previous study [25] that used the CatBoost model, using Python 3.9, to predict the HGS in an older population (≥65 years), which reported an R^2^ of 0.71, our XGBoost model, which was developed using a wider age range (≥19 years), achieved R^2^ values of 0.728 and 0.733 using anthropometric variables with and without the five physical activity levels, respectively. This is the highest R^2^ value obtained among all currently available HGS prediction models.

The F-score, a feature importance metric in XGBoost, indicates how frequently each feature (variable) is used to split the data across all trees in the model. This metric allows for an intuitive comparison of the relative importance of each variable. In this study, weight, age, height, waist circumference, and travel activity received the highest F-scores, in descending order. Similar to our previous study [26], anthropometric factors and age were identified as the most important predictors. However, it is important to note that the F-score has limitations. As it simply provides a measure of the number of times a feature is used in tree splitting, it may not reflect the true underlying importance of that variable. Therefore, the F-score should be interpreted with caution.

Despite the strengths of the proposed model, this study has certain limitations. First, it did not include all of the potential confounding variables affecting HGS. Second, although the sample size was larger compared to that used in other studies, it was not representative of the global population as it only included data from one country in Asia. Therefore, there was an inherent bias owing to ethnic homogeneity. Furthermore, the proposed model has not been extensively validated across diverse populations, including different age groups, clinical conditions, and regional or demographic variations. As a result, its generalizability to broader clinical settings remains uncertain. Future research should focus on external validation with larger and more diverse datasets to enhance the model’s robustness and applicability in real-world healthcare environments. To improve clinical adaptability, future models can be optimized by developing customized evaluation criteria for different population groups, such as older adults, sarcopenic patients, and athletes.

In summary, the proposed XGBoost model demonstrated the potential of machine learning for accurately estimating HGS using complex and large datasets. The HGS estimation model proposed in this study offers several clinical applications.

First, through using basic anthropometric and physical activity data, the model enables the estimation of HGS without the need for direct measurement. This is particularly beneficial in situations where direct measurement is challenging, allowing healthcare professionals to efficiently assess muscle strength. Furthermore, integrating the model into electronic health record (EHR/EMR) systems would facilitate automated HGS estimation when using patient data, providing real-time clinical insights. Potential integration with wearable devices (e.g., smartwatches and fitness trackers) could further enable continuous muscle strength monitoring in daily life.

Second, by integrating this model into routine health screening systems, muscle strength can be assessed in large populations, enabling efficient data collection for large-scale research while saving time and costs.

Thirdly, by employing this model during regular consultations or health check-ups, clinicians can monitor changes in a patient’s HGS over time, allowing for the early identification and prevention of conditions. Specifically, this model can be utilized in rehabilitation settings to monitor muscle recovery and to optimize individualized therapy plans. Additionally, it may aid in chronic disease management for conditions such as diabetes, cardiovascular disease, and sarcopenia by enabling regular HGS assessments for disease progression tracking and early intervention.

## 5. Conclusions

In conclusion, the XGBoost model developed in this study demonstrated superior performance than the MLP and PR models for predicting HGS, achieving the highest R^2^ value. This model holds potential for clinical applications, enabling accurate HGS estimation to support early diagnosis, targeted interventions, and personalized goal setting.

## Figures and Tables

**Table 1:** The baseline characteristics of the 21,147 participants.

	Male (*n* = 10,171)	Female (*n* = 10,976)
Age (mean ± std)	49 ± 16	47 ± 15
19–29 years	1505	1580
30–39 years	1829	2059
40–49 years	2038	2479
50–59 years	1915	2293
Over 60 years	2884	2565
Preferred hand (right/left, *n*)	9104/487	9964/433
Height (cm)	171.08 ± 6.58	158.31 ± 6.15
Weight (kg)	71.78 ± 11.50	57.99 ± 9.22
Waist circumference (cm, mean ± std)	86.28 ± 8.86	78.07 ± 9.49
Body mass index (kg/m^2^, mean ± std)	24.47 ± 3.28	23.15 ± 3.50
Grip strength, right/left (kg)	38.48 ± 7.69/37.04 ± 7.28	22.97 ± 4.90/21.83 ± 4.60
Total physical activity (MET/week)	1218.05 ± 2058.86	867.79 ± 1375.57
Vigorous work activity (MET/week)	103.11 ± 1083.08	16.02 ± 316.45
Moderate work activity (MET/week)	161.39 ± 807.60	113.58 ± 780.04
Travel activity (MET/week)	483.88 ± 827.28	484.38 ± 708.66
Vigorous recreation activity (MET/week)	242.75 ± 780.82	105.80 ± 468.35
Moderate recreation activity (MET/week)	230.92 ± 499.50	152.01 ± 383.11

**Table 2:** Results of the performance comparison between the three machine learning models.

Models including only anthropometric variables without five physical activities levels							
		1st PR	2nd PR	3rd PR	4th PR	DL	XGBoost
RMSE	Train dataset	5.645	5.364	5.292	5.256	5.399	5.230
	Test dataset	5.660	5.380	5.355	5.390	5.399	5.388
R^2^	Train dataset	0.537	0.600	0.616	0.622	0.720	0.728
	Test dataset	0.531	0.595	0.602	0.599	0.713	0.714
Models including both anthropometric variables and five physical activities levels							
		1st PR	2nd PR	3rd PR	4th PR	DL	XGBoost
RMSE	Train dataset	5.610	5.321	5.181	4.989	5.399	5.186
	Test dataset	5.631	5.364	5.421	16.624	5.417	5.360
R^2^	Train dataset	0.546	0.609	0.636	0.672	0.723	0.733
	Test dataset	0.541	0.601	0.601	0.119	0.711	0.717

Anthropometric variables: age, sex, weight, height, and waist circumference. Abbreviations: 1st: first order, 2nd: second order, 3rd: third order, 4th: fourth order, PR: polynomial regression model, DL: deep learning model, and XGBoost: eXtreme Gradient Boosting model.

## Data Availability

The dataset used in this study is publicly available at the Korean National Health and Nutrition Examination Survey repository and can be accessed via their official website (https://knhanes.kdca.go.kr) under the specified terms and conditions. We accessed it on 1 February 2024.
